# High-resolution computed tomography findings in hantavirus pulmonary syndrome

**DOI:** 10.1590/0100-3984.2017.50.4e2

**Published:** 2017

**Authors:** Felipe von Ranke

**Affiliations:** 1 Assistant Professor of Radiology at the Universidade Federal Fluminense (UFF), Niterói, RJ, Radiologist and Medical Coordinator at Dimagem - Diagnóstico por Imagem, Nova Iguaçu, RJ, Brazil. E-mail: feliperanke@yahoo.com.br

Despite the high incidence and lethality of hantavirus pulmonary syndrome (HPS), there is limited information in the literature about the high-resolution computed tomography (HRCT) features of the disease. There have been only a few case reports describing the tomographic findings of HPS^(1-3)^, and there have been none presenting HRCT findings in a series of patients. In the previous issue of **Radiologia Brasileira**, Barbosa et al.^(4)^ published an important study on the HRCT findings in eight patients with confirmed HPS.

HPS is considered an emerging zoonotic disease. Humans are infected by hantaviruses after inhalation of aerosolized virus particles from rodent urine, saliva, or dried excreta^(5)^. Hantaviruses are commonly categorized as Old World or New World, depending on the geographic distribution of their rodent reservoirs, and the illnesses resulting from infection with the Old and New World hantaviruses are designated hemorrhagic fever with renal syndrome and HPS, respectively. Hemorrhagic fever with renal syndrome occurs in Europe and Asia, whereas HPS occurs in the Americas^(6)^. In the Americas, HPS is the most serious manifestation of hantavirus infection, being characterized by severe pulmonary involvement that leads to respiratory failure and cardiogenic shock, with a high case-fatality rate^(6,7)^. The case-fatality rate of HPS in Brazil is quite high, ranging from 33% to 100%, depending on the region^(7)^. Most cases occur in the southern and southeastern regions of the country, and the disease primarily affects young adult males during the course of occupational activities, tourism to rural areas, or flooding. The first cases of HPS diagnosed in Brazil occurred in 1993 in the state of São Paulo^(7)^. Since then, approximately 1,500 cases of HPS have been reported in the country^(7,8)^.

The pathogenesis of HPS is believed to be related to the immune response to the virus, which is responsible for endothelial damage and increased capillary permeability, leading to pulmonary edema. Although hantavirus antigen is present in microvascular endothelial cells, the disturbance is basically functional and most patients do not develop alveolar damage^(5)^. The most commonly reported signs and symptoms are acute fever, chills, generalized myalgia, asthenia, headache, and nausea. During the prodromal phase, dyspnea, tachypnea and dry cough are common^(9,10)^. Respiratory manifestations evolve to the cardiopulmonary phase, thus worsening the respiratory distress, including progressive dyspnea. Within a few hours, the patient develops respiratory failure and a dry cough, which can become productive with expectoration of bloody mucus. Along with respiratory distress and failure, the patient will become hypotensive and tachycardic, and cardiovascular chock soon ensues^(7)^. The reported prevalence of hemorrhagic manifestations among patients with HPS in Brazil ranges from 4% to 37.5%^(7)^. When there is clinical suspicion and the epidemiologic history is consistent with the disease, the etiologic diagnosis is made on the basis of serologic tests and detection of the viral genome by reverse transcriptionpolymerase chain reaction^(5)^. Histopathological examination of the lungs can reveal interstitial and alveolar edema, as well as alveolar hemorrhage and mild interstitial pneumonia, characterized by infiltrates of immunoblasts and mononuclear cells^(5)^.

Recent studies in the radiology literature of Brazil have focused on describing the imaging aspects of numerous infectious diseases^(11-16)^. Radiographically, HPS presents as interstitial edema with or without rapid progression to airspace disease in a central or bibasilar distribution^(5)^. On HRCT, the predominant features are bilateral areas of ground-glass opacities and smooth interlobular septal thickening, although the crazy-paving pattern is observed in a minority of cases. Poorly defined small nodules, focal consolidations, peribronchovascular thickening, and pleural effusion can accompany the main findings^(5)^. Such findings are nonspecific, and the differential diagnosis should consider other infectious and noninfectious lung diseases^(2)^. The clinical and radiological differential diagnoses of HPS include dengue and leptospirosis in epidemic areas; other infectious causes of severe respiratory disease, such as influenza and atypical pneumonia; and acute febrile illnesses such as malaria, yellow fever and spotted fever, especially in tropical areas^(5,7)^.

In conclusion, the Barbosa et al.^(4)^ study contributes important information on the patterns of HRCT findings in a series of patients with HPS. Knowledge of the main radiological and tomographic patterns of HPS, taken together with the relevant clinical and epidemiological data, provides a fundamental contribution to the differential diagnosis of HPS, as well as to the management of HPS patients with acute febrile illness and severe respiratory distress syndrome.
